# Social-ecological correlates of accelerometer-measured occupational sitting among Japanese desk-based workers

**DOI:** 10.1186/s12889-019-7782-1

**Published:** 2019-11-08

**Authors:** Satoshi Kurita, Ai Shibata, Kaori Ishii, Mohammad Javad Koohsari, Koichiro Oka

**Affiliations:** 1 Department of Preventive Gerontology, Center for Gerontology and Social Science, National Center for Geriatrics and Gerontology, Obu, Aichi Japan; 2 Faculty Health and Sport Sciences, University of Tsukuba, Tsukuba, Ibaraki Japan; 3 Faculty of Sport Sciences, Waseda University, Tokorozawa, Saitama Japan; 4 Mary MacKillop Institute for Health Research, Australian Catholic University, Melbourne, Victoria Australia; 5 Behavioural Epidemiology Laboratory, Baker Heart & Diabetes Institute, Melbourne, Victoria Australia

**Keywords:** Workplace, Sedentary behavior, Determinants, Environment

## Abstract

**Background:**

Although the main targets for reducing workplace sedentary behavior have been clarified, only a few studies have examined the association between social-ecological factors and workplace sedentary behavior for effective intervention. The present study aimed to examine the social-ecological factors of workplace sedentary behavior among Japanese sedentary workers.

**Methods:**

Participants were recruited via a cross-sectional mail survey targeting randomly sampled 6000 middle-aged people dwelling in Matsuyama-city and Koto-ku in Japan. Participants answered a questionnaire on social-ecological factors, recorded their work time in a diary, and wore a triaxial accelerometer during waking time for 7 consecutive days. Workplace sedentary behavior was measured using accelerometer and was referred to as the work time in the recorded diary. Full-time workers who had mainly sitting work and valid accelerometer data were included in the analysis. Workplace sedentary variables were sedentary breaks per sedentary hour, sedentary time, and ≥ 30 min bouts of sedentary time. The associations between each sedentary variable and social-ecological factors were explored by conducting three multiple linear regression analyses adjusting for sociodemographic and health-related factors.

**Results:**

A total of 227 participants (133 men, mean age 49.9 ± 6.9 years) were included in the analysis. In the overall sample, “typically seeing work colleagues take sedentary breaks” was significantly associated with more sedentary breaks (B [95% confidence interval {CI}=1.40 [0.07 to 2.73]) and shorter ≥30-min bouts of sedentary time (B [95% CI] = −7.08 [−13.75 to −0.40]). “I am motivated to take sedentary breaks” had an unfavorable association with less sedentary breaks (B [95% CI] = −1.36 [−2.61 to −0.12]) and longer sedentary time (B [95% CI] = 4.15 [0.29 to 8.00]). In male workers, “Too stressed to take sedentary breaks” was significantly associated with less sedentary breaks (B [95% CI] = −5.6 [−9.17 to −2.02]).

**Conclusions:**

Seeing work colleagues take sedentary breaks may be important for reducing workplace sedentary behavior. Those who are more sedentary are motivated to take sedentary breaks. Male workers who feel the need to take sedentary breaks at work are more sedentary.

## Background

Excessive sedentary behavior is a risk factor of several chronic diseases such as cardiovascular disease, stroke, some cancers, and musculoskeletal diseases [1, 2]. In high-income countries, sedentary jobs have rather increased along with technology advancement, which induces automation and efficiency, and workplaces have become a setting where excessive sedentary behaviors occur [3]. For example, Japanese sedentary workers dwelling in two urban areas spent 6.4 h performing sedentary work (69.3% of work hours), which was approximately 3 h more than time to perform other more physically active tasks including standing, walking, and physical labor task [4].

To reduce workplace sedentary behavior, several interventions have been employed, and the effects have been revealed [5, 6]. Breaking sedentary time is a widely feasible method of reducing sedentary time [6]. Additionally, to maximize the effects of the intervention, the factors correlated with workplace sedentary behavior have been explored. There have been many studies examining sociodemographic and health-related factors of workplace sedentary behavior [7–14]. Male sex, younger age, level of education, and higher body mass index (BMI) were among the related factors [8]. Another study found that men reported more short physical activity breaks than women during work hours [7]. Moreover, higher educated workers and young women were more likely to spend more work-related sitting time [13].

Although the potential targets for intervention have been clarified, there is lack of evidence supporting the relationship between social-ecological factors and workplace sedentary behavior for effective intervention [7, 10]. Social-ecological framework considers the complex interplay between individual, interpersonal, and environmental (community, organizational, build environmental, and political) factors, which are essential to promote physical activity [15]. For instance, in workplace sedentary behavior, work-specific individual (job type and work engagement), cultural (lunch away from the desk, walking at lunch and face-to-face interaction), physical (personal printer and office type) and organizational factors (sector) were associated with sedentary time [10].

Another study conducted in Australia explored the effects of social-ecological factors for sedentary breaks during working hours and found that awareness toward sedentary break (intrapersonal factor) was associated with increased sedentary breaks during sedentary times [7]. This study targeted only Australian workers; measured sedentary breaks by self-reporting, which contained recall bias; and did not adjust the covariates such as the sociodemographic factors in the analysis; hence, whether the above factors are important for any workers remains controversial. Although the study set only sedentary breaks as the outcome, if the associations between social-ecological factors of workplace sedentary breaks and sedentary time are also determined, the results will not only show increase in sedentary breaks but also reduction in sedentary time.

Therefore, this study aimed to explore the social-ecological correlates of accelerometer-measured workplace sedentary behavior, including sedentary breaks, sedentary time, and prolonged bouts of sedentary time, among Japanese sedentary workers. As reports have shown that there were different correlates among male and female workers [7, 13], overall and sex-stratified analyses were conducted.

## Method

### Study design and procedure

Cross-sectional data of a project that investigated the association between neighborhood environment and sedentary behavior among Japanese adults aged 40–64 years were used in this study. A postal survey was conducted targeting middle-aged people dwelling in Matsuyama City in Ehime Prefecture, Japan, from July to December 2013 and in Koto Ward in Tokyo from April 2014 to February 2015. Details of the data selection was described elsewhere [16]. In brief, invitation letters were sent to 6000 potential participants who were randomly elected from the resident register. Participants who responded to the invitation were asked to fill out an informed consent form, wear an accelerometer, record their activities in a diary, and respond to a questionnaire. The study was approved by the Research Ethics Committee, Waseda University, Japan (2012–269, 2013–264).

A total of 864 participants (final response rate of 14.4%) responded to the invitation letters, and 778 participants finished the data collection. The participants indicated their working status (full time job, part-time job, no job, full-time homemaker, or student) and main occupational task (sitting task or desk work, standing task, walking task, or physical labor task) in the questionnaire. Among 297 participants who had full-time jobs and mainly sitting task or desk work and had valid accelerometer data with no missing variables (*n* = 227) were included in this study.

### Measurement of sedentary behavior and physical activity

Sedentary behavior and physical activity were measured using a triaxial accelerometer, Active style Pro HJA-350IT (Omron Health Care Co., Ltd., Kyoto, Japan). Participants were asked to wear it on the left side of the waist for 7 consecutive days. This accelerometer has been validated for measuring physical activity and sedentary behavior in a controlled laboratory setting [17, 18] and has acceptable criterion-related validity of sedentary variables against activPAL with built-in inclinometer in a free-living setting [19]. The accelerometer can accurately calculate the participants’ sedentary time especially when they have higher sedentary level. Although the accelerometer cannot discriminate between sitting and static standing postures, it can overestimate sedentary breaks without systematic error. One-minute epoch length was employed for data collection. Accelerometer data were processed using an Omron health management software, BI-LINK for physical activity professional edition ver 1.0 and custom software (Custom-written Macro program). Work hours were measured from the time the participants performed the task until the time they finished the task and recorded it in the activity diary. Valid accelerometer data of a work day were defined as ≥75% wear time during work hours [20] excluding ≥60 consecutive 0.9 Metabolic equivalents (METs) with allowance of up to 2 min of ≤1.0 METs. Those who had more than three valid work days were included in the analysis.

The activity intensity thresholds were 0.9–1.5 METs for sedentary behavior, and ≥ 3.0 METs for moderate-to-vigorous intensity physical activity (MVPA). Sedentary time was calculated from the sum of the minutes when the accelerometer measured sedentary time. A break in sedentary time was defined as an interruption in sedentary time, which occurred from a minute identified as sedentary to an adjacent following minute identified as not sedentary. Sedentary bout was defined as the start and end of the sedentary time period [21]. Sedentary variables were expressed as total sedentary time (%wear time), ≥30-min bouts of sedentary time (%sedentary time), and breaks in sedentary time (times per sedentary hour).

### Social-ecological factors

Social-ecological factors including individual factors, social factors, and work environmental factors were assessed using eight statements with a four-point response scale (Additional file 1) adapted from a questionnaire developed by a previous study conducted in Australia [7]. The original questionnaire comprised 13 statements with a five-point response scale [7]. However, three of the researchers (KO, AS, and KI) modified the questionnaire to make it more applicable to the Japanese workplace and midpoint response style of the Japanese people [22]. These included five statements related to individual factors (“Don’t have enough time to take sedentary breaks,” “Don’t have enough energy to take sedentary breaks,” “Sedentary breaks are a low priority,” “Too stressed at work to take sedentary breaks,” and “I am motivated to take sedentary breaks”), two statements related to social factors (“I typically see work colleagues take sedentary breaks” and “Company should encourage short breaks”), and one statement related to work environmental factor (“There is limited space available at my workplace for me to take a short physical activity break”). All questions were answered using a four-point Likert scale (strongly agree to strongly disagree), which was dichotomized into agree/strongly agree and disagree/strongly disagree for the analysis.

### Sociodemographic factors

Sociodemographic information, including age, sex, and area of residence (Matsuyama City; Koto ward), were obtained from the basic resident register. Other factors were obtained using a self-report questionnaire: education level (high school or lower, 2-year college, or university degree or higher education), household income (<5 million, ≥5 million to <7 million, ≥7 million to <10 million, or ≥10 million), and marital status (currently single or married).

### Health-related factors

BMI and weekly MVPA were used as health-related factors. BMI was calculated from the self-reported height and weight and dichotomized into normal weight (<25.0 kg/m^2^) and overweight and obese (≥25.0 kg/m^2^) considering the imprecision of self-reporting. Weekly MVPA was calculated from the accelerometer data by weighted average of workday and non-work day ([5 × work day + 2 × non-work day]/7).

### Statistical analysis

Descriptive statistics of sociodemographic factors, health-related factors, and sedentary variables were summarized. In order to interpret how sedentary variables, which were continuous variables, change according to each social-ecological factor, multiple linear regressions with forced entry method were conducted, and the linear associations between social-ecological factors and sedentary variables were explored. Individual, social, and work environmental factors were included as independent variables in the models, and sociodemographic and health-related factors were included as adjusted variables. Unstandardized regression coefficient (B) and 95% confidence intervals (95% CI) of each factor for sedentary variables were calculated. Multicollinearity was not observed in any factor. Statistical significance was set at a level of 0.05. All analyses were conducted using IBM SPSS Statistics 25.

## Results

The characteristics of 227 participants (mean age: 49.9 ± 6.9 years, men: 58.6%) are summarized in Table 1. More than half of the participants lived in Koto Ward (58.6%) and were highly educated (56.8% had university degree or higher education). The mean breaks in sedentary time, total sedentary time, and ≥ 30-min bouts of sedentary time during work hours were 8.5 ± 4.4 times, 69.8 ± 13.7% wear time, and 31.5 ± 21.5% wear time, respectively. There were significant sex differences in these sedentary variables (all *p* < 0.01), which indicated that male workers had more sedentary behavior than female workers.

**Table 1:** Characteristics of participants and sedentary variables during work hours

		Total (*n* = 227)	Male (*n* = 133)	Female (*n* = 94)	*p* for sex difference^a^
Socio-demographic factors					
Age		49.9 ± 6.9	50.5 ± 7.2	49.1 ± 6.5	0.15
Area of residence	Matsuyama City	94 (41.4)	57 (42.9)	37 (39.4)	0.60
	Koto Ward	133 (58.6)	76 (57.1)	57 (60.6)	
Education	High school or lower	56 (24.7)	29 (21.8)	27 (28.7)	<0.01
	College	42 (18.5)	14 (10.5)	28 (29.8)	
	University or higher	129 (56.8)	90 (67.7)	39 (41.5)	
Income	< 5 million	79 (34.8)	31 (23.3)	48 (51.1)	<0.01
	≥5 million to <7 million	40 (17.6)	21 (15.8)	19 (20.2)	
	≥7 million to <10 million	58 (25.6)	44 (33.1)	14 (14.9)	
	≥10 million	50 (22)	37 (27.8)	13 (13.8)	
Marital status	Single	58 (25.6)	17 (12.8)	41 (43.6)	<0.01
	Married	169 (74.4)	116 (87.2)	53 (56.4)	
Health-related factors					
BMI	<25 kg/m^2^	172 (75.8)	92 (69.2)	80 (85.1)	<0.01
	≥25 kg/m^2^	55 (24.2)	41 (30.8)	14 (14.9)	
Weekly average MVPA		6.7 ± 3.1	6.9 ± 3.2	6.5 ± 2.8	0.33
Sedentary variables during work hours					
Sedentary time (%wear time)		69.8 ± 13.7	72.0 ± 13.0	66.8 ± 14.2	<0.01
≥ 30-min bouts of sedentary time (% sedentary time)		31.5 ± 21.5	35.7 ± 20.4	25.5 ± 21.7	<0.01
Sedentary breaks per sedentary hour		8.5 ± 4.4	7.7 ± 3.9	9.7 ± 4.7	<0.01

Values were expressed as n (%) or mean ± SD

*BMI* Body mass index, *MVPA* Moderate-to-vigorous physical activity

^a^Categorical variables and continuous variables were compared using the χ^2^ test and ANOVA, respectively

The associations between social-ecological factor and sedentary variables in the multiple linear regression models are summarized in Table 2 for sedentary breaks per sedentary hour. The sedentary break of the overall sample was positively associated with “Typically seeing work colleagues take sedentary breaks” [B (95% CI) = 1.40 (0.07 to 2.73)] and negatively associated with “I am motivated to take sedentary breaks” [B (95% CI) = − 1.36 (− 2.61 to − 0.12)]. For male workers, “Too stressed to take sedentary breaks” was significantly associated with less sedentary breaks [B (95% CI) = − 5.6 (− 9.17 to − 2.02)], while for female workers, “I am motivated to take sedentary breaks” was significantly associated with less sedentary breaks [B (95% CI) = − 2.37 (− 4.6 to − 0.13)].

**Table 2:** Multiple linear regression analyses on the contribution of social ecological factors to sedentary breaks per sedentary hour

	Overall		Male		Female	
	B (95% CI)	*p*	B (95% CI)	*p*	B (95% CI)	*p*
Social ecological factors						
Don’t have enough time to take sedentary breaks	− 0.29 (− 2.03 to 1.46)	0.74	1.81 (− 0.7 to 4.31)	0.16	−0.66 (− 3.65 to 2.32)	0.66
Don’t have enough energy to take sedentary breaks	− 1.61 (− 5.76 to 2.55)	0.45	−4.69 (−12.06 to 2.68)	0.21	−1.27 (−7.31 to 4.78)	0.68
Sedentary breaks are a low priority.	−0.89 (− 2.31 to 0.52)	0.22	−0.68 (− 2.29 to 0.93)	0.40	−1.13 (−3.98 to 1.72)	0.43
Too stressed at work to take sedentary breaks	−1.14 (−3.18 to 0.91)	0.27	**−5.6 (−9.17 to − 2.02)**	**<0.01**	−0.29 (−3.5 to 2.93)	0.86
I am motivated to take sedentary breaks.	**−1.36 (−2.61 to − 0.12)**	**0.03**	−0.85 (−2.36 to 0.66)	0.27	**−2.37 (−4.6 to − 0.13)**	**0.04**
I typically see work colleagues take sedentary breaks.	**1.4 (0.07 to 2.73)**	**0.04**	1.03 (−0.53 to 2.58)	0.19	1.55 (−0.89 to 3.99)	0.21
The company should encourage sedentary breaks.	−0.67 (−2.35 to 1)	0.43	−0.1 (−2.11 to 1.9)	0.92	−0.89 (−3.97 to 2.19)	0.57
There is limited space available at my workplace for me to take a short physical activity break.	0.33 (−1.09 to 1.74)	0.65	−0.37 (−2.14 to 1.39)	0.67	0.37 (−2.22 to 2.96)	0.78
Sociodemographic and health factors						
Sex (ref: male)	**2.13 (0.91 to 3.34)**	**<0.01**				
Age	0.01 (−0.08 to 0.09)	0.90	−0.06 (− 0.15 to 0.03)	0.22	0.12 (−0.05 to 0.28)	0.16
Residence area (ref: Koto-ku)	**2.31 (1.06 to 3.55)**	**<0.01**	**2.71 (1.18 to 4.24)**	**<0.01**	2.13 (−0.17 to 4.42)	0.07
Education (ref: high school or lower)	−0.47 (−1.15 to 0.20)	0.17	−0.45 (−1.25 to 0.36)	0.27	−0.36 (−1.66 to 0.94)	0.58
Income (ref: <5 million)	−0.22 (− 0.78 to 0.35)	0.45	−0.36 (−1.01 to 0.3)	0.28	0.15 (−0.98 to 1.28)	0.80
Marital status (ref: single)	1.24 (−0.16 to 2.65)	0.08	0.38 (−1.57 to 2.33)	0.70	1.02 (−1.31 to 3.36)	0.39
BMI (ref: <25.0 kg/m^2^)	**−1.76 (−3.02 to − 0.49)**	**0.01**	**−2.18 (−3.54 to − 0.82)**	**<0.01**	− 1.13 (−4.02 to 1.76)	0.44
Weekly MVPA	**0.19 (0.00 to 0.38)**	**0.04**	0.13 (−0.07 to 0.33)	0.21	0.36 (−0.04 to 0.77)	0.08
Adjusted *R*^*2*^	0.17		0.23		0.02	

Social-ecological factors were entered as dichotomized variables: ‘disagree/strongly disagree (=1)’ and ‘agree/strongly agree (=2)’

*B* Unstandardized regression coefficient; *BMI* Body mass index, *MVPA* Moderate-to-vigorous physical activity

In total sedentary time and ≥ 30-min bouts of sedentary time, opposite results regarding sedentary breaks were observed (Tables 3 and 4). For overall sample, “I am motivated to take sedentary breaks” and “Typically seeing work colleagues take sedentary breaks” were respectively associated with longer total sedentary time [B (95% CI) = 4.15 (0.29 to 8.0)] and shorter ≥30-min bouts of sedentary time [B (95% CI) = − 7.08 (− 13.75 to − 0.40)]. In male workers, too stressed to take sedentary break was significantly associated with longer total sedentary time [B (95% CI) = 18.65 (7.04 to 30.25)] and ≥ 30-min bouts of sedentary time [B (95% CI) = 34.79 (15.48 to 54.09)]. In addition, male workers who reported that they did not have enough time to take sedentary breaks had significantly shorter sedentary time [B (95% CI) = − 8.32 (− 16.46 to − 0.19)].

**Table 3:** Multiple linear regression analyses on the contribution of social-ecological factors to total sedentary time

	Overall		Male		Female	
	B (95% CI)	*p*	B (95% CI)	*p*	B (95% CI)	*p*
Social ecological factors						
Don’t have enough time to take sedentary breaks	1.02 (− 4.40 to 6.44)	0.71	**−8.32 (−16.46 to − 0.19)**	**0.04**	5.31 (− 3.45 to 14.07)	0.23
Don’t have enough energy to take sedentary breaks	4.81 (−8.09 to 17.71)	0.46	17.25 (−6.69 to 41.2)	0.16	2.15 (−15.61 to 19.92)	0.81
Sedentary breaks are a low priority	2.18 (−2.21 to 6.57)	0.33	1.91 (−3.33 to 7.14)	0.47	0.99 (−7.37 to 9.35)	0.81
Too stressed at work to take sedentary breaks	5.12 (−1.23 to 11.46)	0.11	**18.65 (7.04 to 30.25)**	**<0.01**	4.99 (−4.45 to 14.44)	0.30
I am motivated to take sedentary breaks.	**4.15 (0.29 to 8.00)**	**0.04**	3.52 (−1.38 to 8.43)	0.16	5.52 (−1.05 to 12.09)	0.10
I typically see work colleagues take sedentary breaks.	−2.95 (−7.08 to 1.18)	0.16	−1.9 (−6.95 to 3.15)	0.46	−3.78 (−10.95 to 3.4)	0.30
The company should encourage sedentary breaks.	2.49 (−2.72 to 7.7)	0.35	1.39 (−5.13 to 7.90)	0.67	2.97 (−6.08 to 12.02)	0.52
There is limited space available at my workplace for me to take a short physical activity break.	−0.18 (−4.57 to 4.2)	0.93	1.83 (−3.9 to 7.57)	0.53	0.95 (−6.66 to 8.55)	0.80
Sociodemographic and health factors						
Sex (ref: male)	**−6.21 (−9.98 to − 2.45)**	**<0.01**				
Age	−0.08 (−0.33 to 0.17)	0.52	0.12 (−0.18 to 0.41)	0.44	−0.46 (− 0.94 to 0.03)	0.06
Residence area (ref: Koto−ku)	**−7.57 (−11.44 to − 3.7)**	**<0.01**	**− 9.21 (− 14.19 to − 4.23)**	**<0.01**	−5.97 (− 12.72 to 0.78)	0.08
Education (ref: high school or lower)	1.8 (−0.29 to 3.9)	0.09	2.09 (−0.53 to 4.71)	0.12	1 (−2.81 to 4.8)	0.60
Income (ref: <5 million)	0.76 (−0.98 to 2.51)	0.39	1.16 (−0.96 to 3.27)	0.28	−0.88 (−4.2 to 2.44)	0.60
Marital status (ref: single)	−4.21 (−8.57 to 0.16)	0.06	−1.56 (−7.89 to 4.78)	0.63	−3.3 (−10.15 to 3.56)	0.34
BMI (Ref: <25.0 kg/m^2^)	**4.88 (0.95 to 8.82)**	**0.02**	**6.19 (1.77 to 10.61)**	**0.01**	2.15 (−6.33 to 10.63)	0.62
Weekly MVPA	**−1.01 (−1.6 to − 0.43)**	**<0.01**	**−0.92 (− 1.57 to − 0.27)**	**0.01**	− 1.17 (−2.36 to 0.02)	0.054
Adjusted *R*^*2*^	0.18		0.26		0.05	

Social-ecological factors were entered as dichotomized variables: ‘disagree/strongly disagree (=1)’ and ‘agree/strongly agree (=2)’

*B* Unstandardized regression coefficient; *BMI* Body mass index,, *MVPA* Moderate-to-vigorous physical activity

**Table 4:** Multiple linear regression analyses on the contribution of social ecological factors to ≥30 min bouts of sedentary time

	Overall		Male		Female	
	B (95% CI)	*p*	B (95% CI)	*p*	B (95% CI)	*p*
Social ecological factors						
Don’t have enough time to take sedentary breaks	−0.96 (−9.72 to 7.79)	0.83	−11.78 (−25.31 to 1.76)	0.09	−0.07 (− 13.57 to 13.44)	0.99
Don’t have enough energy to take sedentary breaks	3.95 (−16.9 to 24.81)	0.71	24.39 (−15.44 to 64.22)	0.23	−2.79 (−30.16 to 24.57)	0.84
Sedentary breaks are a low priority.	3.43 (−3.67 to 10.54)	0.34	3.54 (−5.17 to 12.25)	0.42	6.37 (−6.51 to 19.25)	0.33
Too stressed at work to take sedentary breaks	7.78 (−2.48 to 18.05)	0.14	**34.79 (15.48 to 54.09)**	**<0.01**	2.08 (−12.47 to 16.62)	0.78
I am motivated to take sedentary breaks.	2.91 (−3.33 to 9.14)	0.36	1.33 (−6.83 to 9.49)	0.75	5.77 (−4.35 to 15.9)	0.26
I typically see work colleagues take sedentary breaks.	**−7.08 (− 13.75 to − 0.40)**	**0.04**	− 4.21 (− 12.61 to 4.19)	0.32	−8.89 (− 19.95 to 2.16)	0.11
The company should encourage sedentary breaks.	8.2 (−0.21 to 16.62)	0.06	2.49 (−8.35 to 13.32)	0.65	9.17 (−4.78 to 23.12)	0.19
There is limited space available at my workplace for me to take a short physical activity break.	−1.53 (−8.62 to 5.56)	0.67	2.34 (−7.2 to 11.88)	0.63	−2.29 (−14.01 to 9.43)	0.70
Sociodemographic and health factors						
Sex (ref: male)	**−11.09 (− 17.18 to − 5.01)**	**<0.01**				
Age	−0.15 (−0.56 to 0.25)	0.46	0.16 (−0.33 to 0.65)	0.52	−0.52 (−1.27 to 0.22)	0.17
Residence area (ref: Koto−ku)	**−7.6 (−13.85 to − 1.34)**	**0.02**	**−11.4 (− 19.68 to − 3.12)**	**0.01**	−2.84 (− 13.23 to 7.56)	0.59
Education (ref: high school or lower)	1.95 (−1.43 to 5.33)	0.26	−0.86 (−5.22 to 3.50)	0.70	5.70 (−0.16 to 11.56)	0.06
Income (ref: <5 million)	0.97 (−1.85 to 3.79)	0.50	1.79 (−1.74 to 5.31)	0.32	−0.02 (−5.14 to 5.09)	0.99
Marital status (ref: single)	−5.7 (−12.75 to 1.36)	0.11	2.67 (−7.87 to 13.21)	0.62	−8.84 (−19.41 to 1.73)	0.10
BMI (Ref: <25.0 kg/m^2^)	**8.98 (2.63 to 15.34)**	**0.01**	**10.0 (2.64 to 17.35)**	**0.01**	11.86 (−1.21 to 24.93)	0.07
Weekly MVPA	−0.79 (−1.74 to 0.15)	0.10	−0.82 (−1.9 to 0.26)	0.13	−0.85 (−2.68 to 0.98)	0.36
Adjusted *R*^*2*^	0.13		0.16		0.04	

Social-ecological factors were entered as dichotomized variables: ‘disagree/strongly disagree (=1)’ and ‘agree/strongly agree (=2)’

*B* Unstandardized regression coefficient; *BMI* Body mass index, *MVPA* Moderate-to-vigorous physical activity

## Discussion

This study examined the associations of social-ecological factors with objectively assessed workplace sedentary behavior among Japanese workers who have desk work. The findings showed that some social-ecological factors were significantly associated with not only sedentary breaks but also with other sedentary variables after adjusting for sociodemographic and health-related factors. To our knowledge, only one previous study has examined the associations of social-ecological factors with sedentary break and workplace sedentary behavior [7]. This study expanded the understanding of these associations in the context of a non-Western workplace.

In the overall sample, seeing work colleagues taking sedentary breaks had preferable associations with workplace sedentary breaks. This was also associated with ≥30-min bouts of sedentary time. This finding is consistent with Bennie et al.’s study [7] conducted in women. In a qualitative study, the participants stated that concerns about looking unnaturally or feeling self-conscious were barriers to breaking up sitting and standing time [9]. Some workers find it difficult to stand alone when their colleagues are sitting; therefore, work colleagues taking frequent sedentary breaks may be important to reduce workplace sedentary behavior. In order to provide such opportunities, sedentary breaks in the workplace need to be recommended and habituated at an organization level. For example, as an organizational-level strategy, Hadgraft et al. (2016) proposed some methods that were perceived feasible and acceptable: provision of centralized facilities (e.g., bins, printers), communicating face-to-face, standing during meetings, and so on.

The higher motivation to sedentary breaks was associated with less sedentary breaks and a longer sedentary time. The possible interpretation is that those who have a larger volume of workplace sedentary time may have a motivation to take sedentary breaks. This finding was similar with that of an Australian survey, which reported that those who agreed with the advantages of sitting less were more sedentary at work than those who disagreed with the advantages [8]. Although it is difficult to consider that all workers have the knowledge about the harm of excessive sedentary behavior, some studies reported that a sitting-only work was significantly associated with higher body discomfort compared with sit-stand work [23]. Therefore, body discomfort, which more sedentary workers tend to feel, may motivate workers to take sedentary breaks.

In the sex-stratified analysis, too stressed at work to take sedentary breaks was associated with less sedentary breaks and a longer sedentary time in male workers. Bennie et al. [7] reported similar results between too stressed at work to take sedentary breaks and short physical activity breaks. Male workers may have sitting tasks, which makes it difficult for them to take sedentary breaks, or may not be willing to take breaks at work. In the previous qualitative studies on workplace sedentary behavior, the participants stated the nature of the job as a barrier to reduce sedentary behavior [9, 24]. For example, computer-based work and the pressure of having a heavier workload hindered the reduction in sedentary behavior, which we also observed from our sample. Although there is a need to examine the details of the stress at work that hindered one to take a sedentary break, sit-stand workstation may resolve the problem “too stressed at work to take sedentary breaks” because it enables workers to stand without interrupting their job [25] and does not decrease work productivity [23].

In addition, male workers responded that not having enough time to take sedentary breaks was associated with less sedentary time, which was similar to the reports of Bennie et al.’s study (2011). Our results suggest that such male workers were likely to had less sedentary time than those who felt that they had enough time to take sedentary breaks. Therefore, male workers who felt that they did not have enough time to take sedentary breaks may opt to relatively stand at work. For female workers, no correlates were found, except for the motivation to take sedentary breaks. The adjusted *R*^*2*^ value in the three models for female workers was relatively low (0.02 to 0.05, Tables 2, 3, 4), which suggests that it is difficult to explain the correlation between workplace sedentary behavior of female workers and social-ecological factors. Because previous studies reported some correlates for female workers [7, 13], further research is needed to verify these results.

The strength of the present study was that sedentary behavior and physical activity were objectively assessed using triaxial accelerometers. This method contains no recall bias compared with a self-report assessment tool. Another strength was that the participants were recruited from a randomly sampled population living in two different cities, which allowed the collection of data from various workplaces and occupations. This study had some limitations. First, as a cross-sectional study, the causal relationship between the social-ecological factors and workplace sedentary behavior cannot be detected. Second, the number of female workers was small; hence, it may be insufficient to detect significant correlates. Third, the validity of social-ecological factors cannot be shown because the factors depend on the subjective response, and there were no established criteria to measure validity. The present study assessed temporary subjective response of the factors; therefore, the reliability and reproducibility of the response could not be confirmed.

## Conclusions

The present study found that some social-ecological factors were associated with workplace sedentary behavior among Japanese sedentary workers. Our findings suggest that seeing work colleagues take sedentary breaks is associated with more sedentary breaks at work. Organizational strategies to reduce workplace sedentary behavior may be important. Those who are more sedentary may have the motivation to take sedentary breaks. For male workers, the stress at work that hinders them from taking sedentary breaks remains an issue. Hence, further research using a large sample size and with a prospective design is needed to confirm these findings.

## Supplementary information

**Additional file 1.** Questionnaire of the social-ecological factors for sedentary breaks at work.

## Data Availability

The datasets used in this study may be available upon reasonable request to the corresponding author.

## Abbreviations

B: Unstandardized regression coefficient;
BMI: Body Mass Index;
CI: Confidence intervals
METs: Metabolic equivalents;
MVPA: Moderate-to-Vigorous Physical Activity
