# Supplementary material for: Social-ecological correlates of accelerometer-measured occupational sitting among Japanese desk-based workers
Source: BMC Public Health. 2019 Nov 8;19:1489. doi: 10.1186/s12889-019-7782-1 (PMC6842144; doi:10.1186/s12889-019-7782-1)
Supplement: Supplementary file 1 — Additional file 1. Questionnaire of the social-ecological factors for sedentary breaks at work. [file 12889_2019_7782_MOESM1_ESM.docx]

Additional file1: Questionnaire of the social-ecological factors for sedentary breaks at work

**What do you think about “sedentary breaks” during work? For each of the categories listed below, choose the option that best fits your opinion: “strongly disagree,” “disagree,” “agree,” or “strongly agree,” and circle the number.**

|  | **Strongly**  **disagree** | **Disagree** | **Agree** | **Strongly**  **agree** |
| --- | --- | --- | --- | --- |
| 1. Don’t have enough time to take sedentary breaks | 1 | 2 | 3 | 4 |
| 2. Don’t have enough energy to take sedentary breaks | 1 | 2 | 3 | 4 |
| 3. Sedentary breaks are a low priority | 1 | 2 | 3 | 4 |
| 4. Too stressed at work to take sedentary breaks | 1 | 2 | 3 | 4 |
| 5. I am motivated to take sedentary breaks | 1 | 2 | 3 | 4 |
| 6. I typically see work colleagues take sedentary breaks | 1 | 2 | 3 | 4 |
| 7. Company should encourage short breaks | 1 | 2 | 3 | 4 |
| 8. There is limited space available at my workplace for me to take a short physical activity break | 1 | 2 | 3 | 4 |
